# Utility and Impact of the Implementation of Same-Day, Self-administered Electronic Patient-Reported Outcomes Assessments in Routine HIV Care in two North American Clinics

**DOI:** 10.1007/s10461-022-03585-w

**Published:** 2022-01-22

**Authors:** Duncan Short, Rob J. Fredericksen, Heidi M. Crane, Emma Fitzsimmons, Shivali Suri, Jean Bacon, Alexandra Musten, Kevin Gough, Moti Ramgopal, Jeff Berry, Justin McReynolds, Abigail Kroch, Brenda Jacobs, Vince Hodge, Divya Korlipara, William Lober

**Affiliations:** 1 Global Implementation Science, ViiV Healthcare, 980 Great West Road, Brentford, Middlesex TW8 9GS UK; 2 Center for AIDS Research, University of Washington, Seattle, WA USA; 3 St Michael’s Hospital, Toronto, ON Canada; 4 Ontario HIV Treatment Network, Toronto, ON Canada; 5 Midway Specialty Care Center, Fort Pierce, FL USA; 6 TPAN, Chicago, IL USA

**Keywords:** Quality of life, Implementation science, Patient-reported outcomes, Suicidal ideation, HIV care

## Abstract

The PROgress study assessed the value and feasibility of implementing web-based patient-reported outcomes assessments (PROs) within routine HIV care at two North American outpatient clinics. People with HIV (PWH) completed PROs on a tablet computer in clinic before their routine care visit. Data collection included PROs from 1632 unique PWH, 596 chart reviews, 200 patient questionnaires, and 16 provider/staff questionnaires. During an initial setup phase involving 200 patients, PRO results were not delivered to providers; for all subsequent patients, providers received PRO results before the consultation. Chart review demonstrated that delivery of PRO results to providers improved patient-provider communication and increased the number of complex health and behavioral issues identified, recorded, and acted on, including suicidal ideation (88% with vs 38% without PRO feedback) and anxiety (54% with vs 24% without PRO feedback). In post-visit questionnaires, PWH (82%) and providers (82%) indicated that the PRO added value to the visit.

**Supplementary Information:**

The online version contains supplementary material available at 10.1007/s10461-022-03585-w.

## Introduction

Life expectancy of people with HIV (PWH) has significantly increased over the past 30 years with continuous improvements in antiretroviral therapy, in many regions reaching lengths comparable to the general population [1–3]. This increase has led to a greater focus on overall patient well-being and health-related quality of life as treatment outcome targets [3–5]. Focusing on person-centered healthcare to address a range of determinants of poor health beyond viral suppression will enable PWH to benefit from healthy aging along with ongoing viral suppression [4]. Many symptoms, health behaviors, and life circumstances associated with living with HIV cannot be measured by laboratory values or other direct observation approaches and are often under-addressed in clinical care [6]. Rates of substance use, depression, intimate partner violence, and homelessness are higher among PWH than the general population [7–12], and a better understanding of such issues, as well as other information beyond standard clinical measures, has the potential to improve outcomes for PWH. For example, provider attempts to support PWH to maintain adherence to a treatment regimen can benefit from understanding patient behaviors and risks to adherence including substance use, depression, stigma, life circumstances such as housing status, and treatment satisfaction [13]. Furthermore, knowledge of sexual risk behavior can help identify appropriate opportunities to discuss pre-exposure prophylaxis to prevent HIV transmission [13].

Assessments that evaluate patient-reported measures and outcomes (PROs) have the potential to provide a systematic, effective, and timely detection of clinically relevant issues by gathering patients’ own views and insights before a patient-provider discussion and presenting them in a simple, easily digestible format that can inform action-oriented decision-making in a consultation [4, 13, 14]. A review of 27 oncology studies concluded that well-implemented PROs in the clinical setting had a positive impact on detecting otherwise unrecognized problems, improved the monitoring of treatment responses, and enhanced patient-provider communication and patient satisfaction [14]. In the field of HIV, a small number of studies have shown that the use of PROs in care enhances communication between patients and providers and improves providers’ ability to detect and monitor symptoms and health behaviors that might otherwise be missed, particularly mental health issues and substance use [6, 15, 16]. In a large US cohort of HIV clinics, tablet-based PROs have been well accepted and valued by providers [17] and reported as highly acceptable and easy to use by PWH [18].

The use of PROs in the field of HIV care to date has primarily been restricted to clinical trials, research studies, and large academic centers. Despite the clear benefits of successful PRO implementation within clinical care, the effective integration of PROs into care settings for PWH outside of large, highly resourced sites is uncommon. The PROgress study was conducted at 2 community care clinics in North America and aimed to identify the essential program elements that can support the sustainable implementation of PROs into routine HIV care in these settings and to examine the added value of implementing PROs for salient stakeholders, including PWH, providers, and other clinic staff.

## Methods

### Study Design

The PROgress study was designed and monitored with the support of a steering committee, which included PWH, HIV care providers, clinic directors, and healthcare researchers. The design of the study was informed by a targeted literature review focused on the impact of administering PROs within HIV clinical care [13, 19], which evolved throughout the project duration and is available on the PROgress study website at https://progresshivcare.org/.

The study was a prospective, hybrid type three implementation-effectiveness study conducted with 2 community care outpatient clinics with comparable resources: one within St Michael’s Hospital (SMH), Toronto, Ontario, Canada; the other, the Midway Specialty Care Center (MSCC) in Fort Pierce, Florida, USA, between August 2018 and July 2020. The study sites were selected on the basis of several factors, including the number of providers to experience the intervention, caseload, infrastructure, on-site referral resources (e.g., case management, pharmacy, psychiatry), and PRO interest. The study aimed to gain implementation and effectiveness insights regarding the establishment and use of PROs in the clinics, drawing significantly upon an implementation science framework covering 5 outcome domains: reach, effectiveness, adoption, implementation, and maintenance (RE-AIM) [20]. RE-AIM is an established and widely used evaluation framework for assessing the feasibility, quality, and impact of a health intervention [21]. Acceptability was also included as a focus of this research as this was considered a key outcome of interest relating to future scale-up. The primary objectives of the study (Table 1) were (1) to understand and assess the number, proportion, and representativeness of individuals who are willing and able to successfully engage in the process (reach); (2) to assess the impact of the PRO intervention on the patient-provider interaction, clinic operation, and clinical/medical practice (effectiveness); (3) to assess the number, proportion, and representativeness of clinic personnel adopting the intervention (adoption); (4) to assess the degree to which the fidelity of PRO integration is upheld, including consistency of delivery, use as intended, and the time and cost of the intervention (implementation); (5) to assess the extent to which the intervention can be sustained over the longer term at the setting and individual levels (maintenance); and (6) to assess the acceptability of the intervention from the perspective of the stakeholders (patients, providers, and other clinic staff).

**Table 1:** Primary objectives and data sources for the PROgress study

Objectives	Data sources
*Reach* To understand and assess the number, proportion, and representativeness of individuals who are willing and able to successfully engage in the process	Numbers of PROs initiated and completed
*Effectiveness* To assess the impact of the PRO intervention on the patient-provider interaction, clinic operation, and clinical/medical practice	Chart reviews, one-on-one interviews^a^ and structured surveys with participants (PWH, providers, and clinic staff)
*Adoption* To assess the number, proportion, and representativeness of clinic personnel adopting the intervention	Post-interview structured surveys with participating providers and clinic staff
*Implementation*^b^ To assess the degree to which the fidelity of PRO integration is upheld, including consistency of delivery, use as intended, and the time and cost of the intervention	One-on-one interviews^a^ and post-interview and post-training structured surveys with participating providers and clinic staff Time and cost assessment by designated site coordinators
*Maintenance* To assess the extent to which the intervention can be sustained over the longer term at the setting and individual levels	One-on-one interviews^a^ with participating providers and clinic staff
*Acceptability* To assess the acceptability of the intervention from the perspective of the stakeholders (patients, providers, and other clinic staff)	Acceptability E-scale and one-on-one interviews and structured surveys with participating PWH and providers

*PWH* people with HIV, *PRO* patient-reported outcomes assessment

^a^Qualitative analysis of one-on-one interviews is part of planned future work and is not described here

^b^A secondary objective was to obtain feedback from stakeholders on the wider project implementation strategies

The study was divided into three phases for each site: the scoping and preparation phase (preparation), the PRO setup and testing phase (setup), and the PRO delivery phase (delivery). The preparation phase included PRO selection, planning patient-flow maps, provider/clinic staff training, and outcome summary sheet design. The setup phase included setup and testing of PROs. During this phase, eligible PWH self-administered an electronic PRO assessment on-site immediately before a routine care visit, but the assessment results were not shared with the provider. The delivery phase included integration of the PRO intervention within routine care. During this phase, the results of PRO assessments completed by participating PWH were summarized in an instant succinct report that was hand delivered to the provider before seeing the patient. Therefore, patient-provider consultations during the setup phase were carried out without prior PRO feedback, and consultations during the delivery phase were carried out with prior PRO feedback.

### Participating PWH

The study included adults (aged ≥ 18 years at study entry) with a diagnosis of HIV infection who were attending one of the participating clinics for a routine visit during the study period and who could speak and understand English, Spanish, and/or Haitian Creole sufficiently to be able to complete the PRO assessment. PWH visiting the clinic to see a provider other than their primary HIV care provider or for a non-routine reason, such as acute injury or illness, were not eligible. Those with known acute or significant prohibitive psychiatric, cognitive, or motor impairment or those who appeared acutely intoxicated were excluded.

The planned sample size was 1800 PWH across the two study sites. This was based on attendance estimations over a time period that would enable robust beta testing and integration of PROs within care and the embedding of a process of sufficient length to enable clinic staff to provide valid feedback on implementation.

### Participating Providers and Staff

Providers and clinic staff at each site were invited to participate in a brief survey regarding utility of and satisfaction with PRO implementation.

### Preparation

To anticipate the barriers and facilitators to the successful integration of the PROs, consideration was given to salient implementation constructs, published literature, and the previous experience of PRO integration by members of the team [6, 16, 17, 22]. Implementation strategies were then co-created with the sites, which aligned to the desired study objectives and outcomes (Table 2). The Expert Recommendations for Implementing Change (ERIC) were used to help inform this development [23].

**Table 2:** Implementation strategies derived from reference to the Expert Recommendations for Implementing Change (ERIC) [23]

Implementation strategies	Strategy details
*Use evaluative and iterative strategies* Assess for readiness and identify barriers and facilitators Conduct local needs assessment Develop a site-specific action plan	At the outset, a site visit assessment will be conducted to observe clinic operation and meet with salient pathway stakeholders to promote engagement, identify concerns and challenges (such as capacity barriers), and explore feasible pathway adaptations that will enable integration We will understand the process and logistics of current clinic flow and how the process can be implemented; this includes understanding the patient journey, options for timing of PRO data collection, and any logistical considerations (e.g., printer locations if the provider uses a printout of the data) Ahead of implementation, a site-specific action plan will be created for implementation, including the process/flow and choice of PROs to meet clinic needs
*Adapt and tailor implementation to context* Promote adaptability and integrate the PRO process within the current workflow and clinic operation	The implemented process will be designed to improve patient management and will consider how this can dovetail and improve the current workflow. For example, how can the implementation support or enhance current workflow, such as adopting timings to suit clinic patterns and gathering data that are needed for both clinical and site needs in a more efficient way and not have a detrimental effect on the fidelity of the intervention
*Engaging and involving stakeholders in the process as partners to support implementation* Involve leadership from the outset Involve clinic staff Identify and prepare champions	We will seek buy-in from senior stakeholders to ensure that we have full endorsement and commitment to implement The clinical teams will be involved in the decision-making process regarding the PRO intervention, including the PRO domain content, feedback form design, and levels of risk to flag to minimize the need for incremental iterative changes, which can be time-consuming and cause confusion A role will be defined to champion this process—this may be a research coordinator or similar
*Train and educate stakeholders* Conduct educational meetings Develop educational materials	Educational materials and training will be conducted to ensure that stakeholders understand the value and process of the PRO data collection intervention
*Change infrastructure* Provide IT infrastructure to enable PRO integration	Integrating the process will involve provision of tablet computers with functionality for data collection, the development of a summary sheet of PRO outcomes, and the provision of tablet computers and printers

*IT* information technology, *PRO* patient-reported outcomes assessment

Implementation strategies included readiness and needs assessments, including site visits; engaging and involving stakeholders in the process as partners; changing infrastructure; training of staff; and the development of site-specific action plans.

The initial stages of the preparation phase of the study included the designation of a site research coordinator and the definition of responsibilities for administering, tracking, and responding to PROs (to ensure all steps of the PRO administration were followed for each patient); the development of the electronic PROs (described in more detail in the next section) and patient-flow maps; and the creation of provider and clinic staff training protocols. Staff received on-site training relating to the integration of the PROs into clinical practice, conducted by three study investigators with expertise in implementing PROs (H Crane, R Fredericksen, and W Lober). To maintain fidelity of the trainers, all three study investigators were present for trainings at each site, and trainings were conducted using a uniform presentation developed by these investigators. Hardware (tablet computers/iPads) and logistical systems were introduced and tested, and any changes deemed necessary to improve implementation efforts were made.

### PRO Content and Administration

**Fig. 1:**
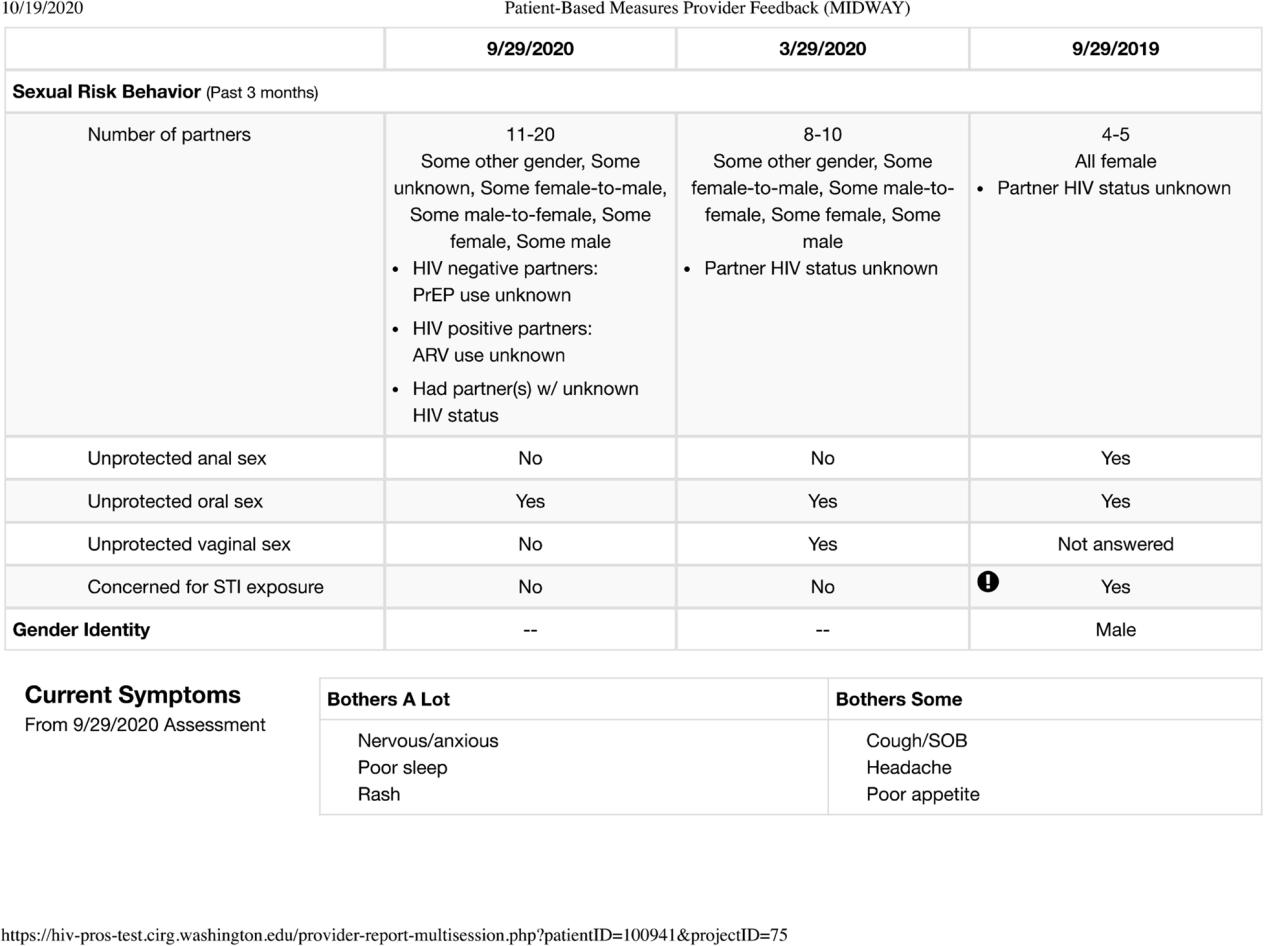
Example of patient-reported outcomes assessment provider feedback summary (based on a fictional patient). *AUDIT-C* Alcohol Use Disorders Identification Test for Consumption, *DOB* date of birth, *HATQoL* HIV/AIDS-Targeted Quality of Life, *IPV* intimate partner violence, *MRN* medical record number, *PHQ-9* Patient Health Questionnaire 9

PROs were administered using a previously developed PRO platform (http://cprohealth.org) and included a selection of PRO instruments that were agreed upon by each site in conjunction with the study sponsor and the evaluation team from the University of Washington. Common, validated, widely used PRO instruments (e.g., PHQ-9 for depression, AUDIT-C for alcohol use) were proposed to clinic leadership and providers for inclusion. Additional measures were chosen based on individual clinic population needs and/or research interest, for example, SMH included measures on Canadian immigration status, sexual behavior under the influence of substances, and health service utilization. PRO instruments were selected for their ability to provide clinically actionable information that could inform decision-making at the time of the consultation. The PRO instruments selected by the study sites covered several clinical domains including antiretroviral adherence, substance use, depression/suicidal ideation, sexual risk behavior, and intimate partner violence (Table 3). Skip logic was applied to reduce the burden of questions for patients where applicable; thus, the number of questions included in the assessments ranged from 65 to 101 at MSCC and from 51 to 100 at SMH. The PRO assessment was administered in electronic format on a tablet computer with touch-screen entry. This form of data capture minimizes the risk of data entry and transfer errors and is also easier for patients as it does not require the use of a mouse or keyboard. Completed PROs were scored using automated algorithms, based on the PRO instrument developers’ instructions, and the results were summarized in a 1-page printed report that was shared with the provider immediately before the clinic visit (Fig. 1) and could be subsequently uploaded or added to the medical record. MSCC scanned the PRO results report into the electronic medical record at the end of each day. SMH did not incorporate PRO results reports into medical records. Eligibility for future follow-up assessments within the PRO platform was set for a minimum of 105 days, to minimize potential clinic burden and/or patient survey fatigue, while still capturing changes in key domains (e.g., depression, ART adherence, substance use) in a timely fashion; results from follow-up assessments are not reported here.

**Table 3:** Patient-reported outcomes assessment domains and instruments included in PROgress

Tool	MSCC	SMH
Housing	Yes	Yes
Nutrition (Canadian Nutrition Screening Tool) [33]	Yes^a^	Yes
Depression (PHQ-9) [34, 35]	Yes	Yes
Anxiety (HIV symptoms inventory) [36]	Yes	Yes
Adherence (VAS)	Yes	Yes
Satisfaction with HIV medications (HATQoL) [37]	Yes	Yes
Nicotine use	Yes	Yes
Alcohol use (AUDIT-C) [38]	Yes	Yes
Substance use (ASSIST) [39]	Yes	Yes
Gender identity	Yes	Yes
Sexual risk behavior (SRBI) [40]	Yes	Yes
Sexual orientation	Yes	Yes
Intimate partner violence (IPV-4) [41]	Yes	Yes
Acceptability E-scale [18]	Yes	Yes
Other current healthcare	No	Yes
Affordability of medication	No	Yes
Sex-enhancing substance use, “Chemsex”	No	Yes

*ASSIST* Alcohol, Smoking and Substance Involvement Screening Test, *AUDIT-C* Alcohol Use Disorders Identification Test for Consumption, *HATQoL* HIV/AIDS-Targeted Quality of Life, *IPV-4* Intimate Partner Violence-4, *MSCC* Midway Specialty Care Center, *PHQ-9* Patient Health Questionnaire 9, *SMH* St Michael’s Hospital, *SRBI* Sexual Risk Behavior Inventory, *VAS* visual analog scale

^a^At MSCC, only the weight loss item was included

### Assessments and Outcomes

Evaluation tools used in the study are summarized in Table 4. To understand and assess the number, proportion, and representativeness of PWH who were willing and able to successfully engage in the process (reach), quantitative data were collected on a screening form and in the PRO platform itself. Data included reasons for not completing the assessment, percentage of PWH progressing through the PRO process, and percentage of PWH requiring or requesting support to complete the PRO process. An incomplete PRO was defined as “not advancing beyond sexual risk behavior items”; these items are approximately 75% of the way through the assessment.

**Table 4:** Overview of evaluation tools used in the PROgress study

Evaluation tool/method	Study phase	Administered	Participants	n	Measured	Remuneration
PRO training evaluation	Preparation	At conclusion of training	Staff and providers	18	Perceived quality of PRO training	None
Acceptability E-scale survey	Setup and delivery	Within PRO assessment	Patients	1102	Acceptability and usability of PRO platform	None
PIPPI survey	Delivery	On-site, after care visit	Patients	200	Perceived impact of PROs on patient-provider communication	US $10
Brief clinical/non-clinical staff post-interview survey	Delivery	At conclusion of one-on-one interview	Staff and providers	16	Perceived utility of PROs in practice	None
Chart review	Setup and delivery	NA, performed by on-site research coordinator	Patients	596	Incidence of provider documentation of issues identified by PRO and subsequent referral	NA

*NA* not applicable, *PIPPI* perceived impact of PROs on patient-provider interaction, *PRO* patient-reported outcomes assessment

To determine the frequency of discussion of symptoms and behaviors identified by the PRO and the frequency of referral to other care providers on the basis of PRO information (effectiveness), chart reviews of medical records were carried out for same-day clinic visits for participating PWH who completed a PRO in the setup phase (without delivery of PRO feedback to providers; N = 200, 100 per site) and in the delivery phase (with delivery of PRO feedback to providers; N = 396, 199 MSCC, 197 SMH). Chart reviews evaluated discussion and referral of depressive symptoms/suicidal ideation, anxiety, antiretroviral medication adherence and satisfaction, substance use, alcohol use, intimate partner violence (IPV), sexual risk behavior, and nutrition (measures listed in Table 3).

Perspectives of participating PWH on the acceptability and usability of the PRO platform were evaluated by means of the Acceptability E-scale incorporated into the PRO (Supplementary Appendix 1), which measures 7 components of acceptability using Likert scales [18]. This was supplemented by 200 post-visit structured surveys carried out during the delivery phase to assess patient perception of the impact of PROs on care (100 per site; Supplementary Appendix 1). Data on the time spent on completion of PROs were provided from the PRO platform. Time spent on the informed consent process was not included in the calculation of duration of PRO completion time. Perspectives of participating providers and non-medical clinic staff on the utility and value added of integrating PROs into care were evaluated by means of structured surveys (n = 16), including statements for which participants were asked to indicate their level of agreement using Likert scales (Supplementary Appendix 1). Assessment of the quality and usefulness of training during the preparation phase was conducted via a post-training structured survey (Supplementary Appendix 1) administered to staff and providers in attendance (n = 18).

### Informed Consent Procedures

Participating PWH provided written informed consent on-site at the time of their care visit before each study activity. There was no remuneration for completing the PRO. For the post-visit structured survey, patients were paid US $10 for their participation. Providers and staff provided written informed consent before all study activities; verbal consent was used for the post-training evaluation. Providers and staff were invited to participate in study activities by an on-site research coordinator and were not remunerated for participation. Approval for research involving human participants was granted for all study activities through the SMH Research Ethics Board and through the University of Washington Institutional Review Board for MSCC.

### Statistical Analysis

Statistical analyses were performed using STATA version 14.2 (StataCorp LLC, College Station, TX). Data presentation is descriptive, with mean and standard deviation for continuous variables and percent distribution for categorical variables. Comparison of the chart reviews relating to phases when providers did and did not receive the PRO results used the Fisher exact test with a significance level of 0.05.

## Results

### Study Population

**Fig. 2:**
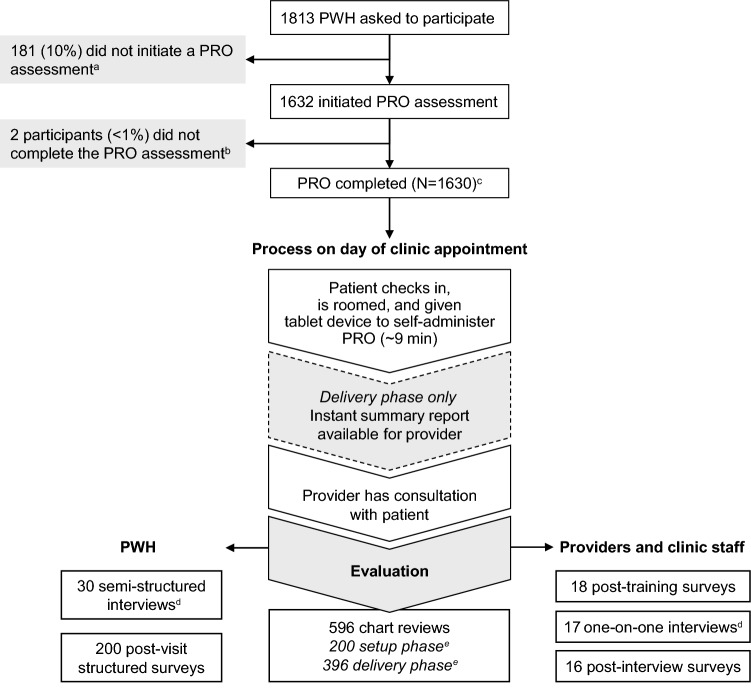
PROgress study design and participants. *PWH* people with HIV, *PRO* patient-reported outcomes assessment. ^a^Reasons for not initiating the PRO were language barriers (n = 68), felt not needed/useful (n = 22), literacy barriers (n = 21), not in mood (n = 13), vision problems (n = 11), perceived length (n = 10), cognitive problems (n = 7), too sick (n = 3), tired of it (n = 1), other reason (n = 6), and no reason given (n = 19). ^b^2 participants did not complete the PRO for unknown reasons. ^c^Including 1102 completed Acceptability E-scales. ^d^Qualitative analysis of one-on-one interviews is part of planned future work and is not described here. ^e^Setup phase = without PRO feedback to providers; delivery phase = with PRO feedback to providers

A total of 1813 eligible PWH were asked to participate in the study, 1632 (90%) initiated a PRO assessment, and of those, 1630 (> 99%) completed the assessment (Fig. 2). The Acceptability E-scale was completed by 1102 PWH.

**Fig. 3:**
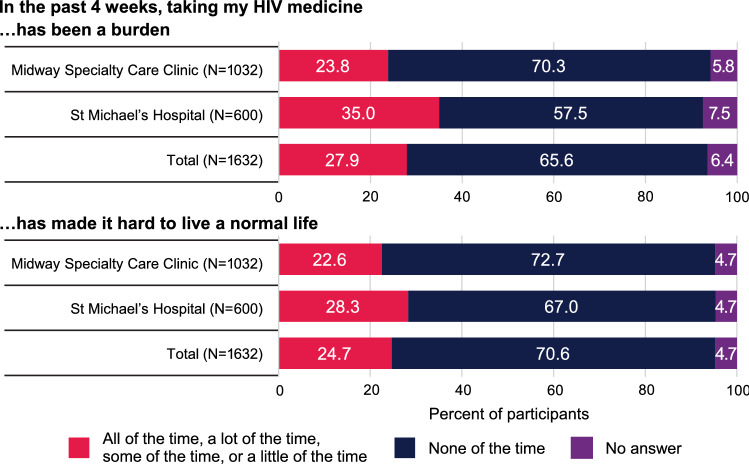
Patient-reported outcomes assessment responses to HIV/AIDS-Targeted Quality of Life satisfaction with HIV medications questions

Demographic data for PWH included in the chart reviews showed that the study population was reflective of the overall clinic populations, and demographic characteristics of participating PWH in the setup and delivery phases were similar (Table 5). Most participating PWH were men, and approximately half were aged ≥ 50 years (Table 5). Anxiety and depression were commonly reported (34% and 27% of participating PWH, respectively), and 7% of participating PWH reported having suicidal ideation on “more than half the days” or “nearly every day” (SMH) or on “several days,” “more than half the days,” or “nearly every day” (MSCC) over the previous 2 weeks (Table 5). One-quarter (25%) of PWH included in the chart reviews indicated that they were dissatisfied with their HIV medication, responding “some of the time” or more often on one or both HIV/AIDS-Targeted Quality of Life instrument (HATQoL) items “…taking my [HIV] medicine has been a burden” and “…taking my [HIV] medicine has made it hard to live a normal life” (Table 5). This level of dissatisfaction was consistent with results from the total study population (Fig. 3).

**Table 5:** Demographic characteristics, symptoms, and behaviors of participating PWH included in the chart reviews and post-visit structured surveys

Parameter, n (%)	Chart reviews			Post-visit survey (N = 200)
	Setup phase (N = 200)	Delivery phase (N = 396)	Total (N = 596)	
Age				
< 30	24 (12)	44 (11)	68 (11)	16 (8)
30 to < 40	34 (17)	83 (21)	117 (20)	37 (18)
40 to < 50	35 (18)	74 (19)	109 (18)	41 (20)
50 to < 60	50 (25)	85 (21)	135 (23)	50 (25)
≥ 60	55 (28)	110 (28)	165 (28)	56 (28)
Sex at birth				
Male	137 (68)	272 (69)	409 (69)	144 (72)
Female	63 (32)	124 (31)	187 (31)	56 (28)
Race				
Black	83 (42)	171 (43)	254 (43)	81 (40)
White	79 (40)	153 (39)	222 (37)	76 (38)
Asian	9 (4)	15 (4)	24 (4)	10 (5)
Other/NS^a^	29 (15)	57 (14)	86 (14)	33 (17)
Ethnicity				
Hispanic	13 (6)	29 (7)	42 (7)	11 (6)
Symptoms and behaviors				NR
Dissatisfaction with ART^b^	54 (27)	97 (24)	151 (25)	
ART adherence ≤ 90%	36 (18)	58 (15)	94 (16)	
Any IPV	25 (13)	24 (6)	49 (8)	
Suicidal ideation	13 (7)	26 (7)	39 (7)	
Anxiety	62 (31)	142 (36)	204 (34)	
Depression	61 (31)	99 (25)	160 (27)	
Nutrition (CNST)^c^	85 (43)	147 (37)	232 (39)	
Hazardous drinking (AUDIT-C)	40 (20)	107 (27)	147 (25)	
Substance use (ASSIST)	24 (12)	57 (14)	81 (14)	

*ART* antiretroviral therapy, *ASSIST* Alcohol, Smoking and Substance Involvement Screening Test, *AUDIT-C* Alcohol Use Disorders Identification Test for Consumption, *CNST* Canadian Nutrition Screening Tool, *HATQoL* HIV/AIDS-Targeted Quality of Life, *IPV* intimate partner violence, *NR* not recorded, *NS* not specified, *PWH* people with HIV, *PRO* patient-reported outcomes assessment, *ROS* review of systems

^a^Including aboriginal, First Nations, Middle Eastern, mixed race, and Native American

^b^Responded “some of the time” or greater on one or both of the following HATQoL items: “…taking my [HIV] medicine has been a burden” and “…taking my [HIV] medicine has made it hard to live a normal life”

^c^Weight loss without trying on CNST or ROS item 177 re weight loss, or 176 re weight gain/fat

Participating providers taking part in the delivery phase survey included 5 medical doctors, 1 nurse practitioner, 1 physician’s assistant, 2 pharmacists, and 2 registered nurses. Five non-medical clinic staff also completed delivery phase surveys. The post-training survey was completed by 18 clinic staff (specific roles were not captured in the survey).

### ‘Reach’—PWH Willing and Able to Successfully Engage with PROs

Among 1813 eligible PWH asked to participate, 1632 (90%) provided informed consent and began the PRO assessment. Reasons for the 181 refusals or failures to begin the assessment included language barriers (n = 68, 38%), literacy barriers (n = 21, 12%), and vision problems including those related to availability of spectacles (n = 11, 6%). Twenty-two PWH (12%) felt that the PRO assessment was not needed or was not useful (Supplementary Fig. 1). All but 2 of the participating PWH completed the PRO assessment. The mean time taken to complete the PRO assessment was 9 min across both sites.

### ‘Effectiveness’ of PROs on Patient–Provider Interaction, Clinic Operation, and Clinical/Medical Practice

#### Impact of PROs on Clinical/Medical Practice Outcomes

Delivery of the PRO assessment to the provider in advance of the patient’s visit increased the number of complex health and behavioral issues that were identified, recorded, and acted on. Comparison of chart reviews from the preparation phase (without PRO feedback to providers) and the delivery phase (with PRO feedback to providers) showed that when provided with PRO assessment data in advance, providers were significantly more likely to document suicidal ideation (*P* = 0.002) and anxiety (*P* < 0.001) and significantly more likely to refer to mental health services for anxiety (*P* = 0.008; Table 6). Other notable increases in provider documentation that did not reach statistical significance, but merit further investigation, include dissatisfaction with antiretroviral medication, depression, and having experienced psychological violence (Table 6). There were no differences between sites in rates of documentation (data not shown).

**Table 6:** Impact of PROs on provider behavior: documentation of symptoms, behaviors, and referrals in the setup phase (without PRO feedback to providers, N = 200) and delivery phase (with PRO feedback to providers, N = 396)

Symptom/behavior	PRO feedback to providers^a^	N^b^	Documentation		Referral	
			n (%)^c^	*P* value^d^	n (%)^c^	*P* value^d^
Any IPV^e^	Without	25	8 (32)	0.77	NA	NA
	With	24	9 (38)		NA	
Sexual risk behavior^f^	Without	14	4 (29)	1.0	NA	NA
	With	20	7 (35)		NA	
Adherence ≤ 90%	Without	36	30 (83)	0.77	NA	NA
	With	58	50 (86)		NA	
Dissatisfaction with ART regimen (HATQoL)^g^	Without	54	23 (43)	0.09	NA	NA
	With	97	56 (58)		NA	
Suicidal ideation^e,h^	Without	13	5 (38)	0.002	7 (54)	0.48
	With	26	23 (88)		18 (69)	
Anxiety^h^	Without	62	15 (24)	< 0.001	11 (18)	0.008
	With	142	77 (54)		52 (37)	
Depression^h^	Without	61	26 (43)	0.08	19 (31)	0.40
	With	99	57 (58)		38 (38)	
Nutrition (CNST)^h,i^	Without	85	31 (36)	0.58	31 (36)	0.58
	With	147	60 (41)		60 (41)	
Hazardous drinking (AUDIT-C)^h^	Without	40	12 (30)	0.40	6 (15)	0.38
	With	107	25 (23)		10 (9)	
Substance use (ASSIST)^h^	Without	24	7 (29)	0.80	3 (12)	1.0
	With	57	20 (35)		7 (12)	

*ART* antiretroviral therapy, *ASSIST* Alcohol, Smoking and Substance Involvement Screening Test, *AUDIT-C* Alcohol Use Disorders Identification Test for Consumption, *CNST* Canadian Nutrition Screening Tool, *HATQoL* HIV/AIDS-Targeted Quality of Life, *IPV* intimate partner violence, *NA* not applicable, *PRO* patient-reported outcomes assessment, *ROS* review of systems

^a^Setup phase = without PRO feedback to providers; delivery phase = with PRO feedback to providers

^b^Symptom endorsed in PRO assessment

^c^Percent of total endorsed

^d^Fisher exact test

^e^Providers were alerted in both phases

^f^At St Michael’s Hospital only

^g^Responded “some of the time” or greater on 1 or both of the following HATQoL items: “…taking my [HIV] medicine has been a burden” and “…taking my [HIV] medicine has made it hard to live a normal life”

^h^Referrals were counted as discussions

^i^Weight loss without trying on CNST or ROS item 177 re weight loss, or 176 re weight gain/fat

#### Providers’ Experience of Using PROs and Perception of the Impact of PROs on Their Clinical Practice

**Fig. 4:**
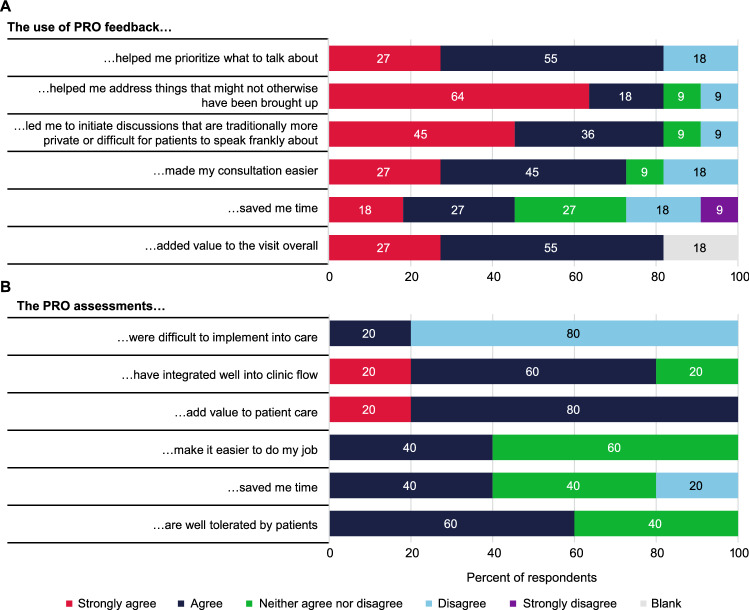
Results of structured surveys carried out during the delivery phase with **A** providers (N = 11) and **B** clinic staff (N = 5) to assess the perceived impact of PROs. *PRO* patient-reported outcomes assessment

The delivery phase structured surveys with providers indicated that PROs helped illuminate patient needs and issues that are often not observable and/or not adequately addressed in consultations. Among 11 providers, 9 (82%) either agreed or strongly agreed that the PROs helped them prioritize discussion points with the patient, identified topics that might otherwise not have been brought up, led to more discussions on potentially sensitive topics, and added value to the visit overall (Fig. 4A). Most providers (8/11, 73%) also found that the PROs made their consultation easier.

#### Providers’ Perceived Impact of the Process Upon Their Consultation Workload and Time

The PROs were regarded as having a minimal and/or manageable impact upon workload and time for providers. Survey results showed some disagreement on whether PROs saved time during the consultation: 5 (45%) agreed, 3 (27%) disagreed, and 3 (27%) neither agreed nor disagreed.

#### Clinic Staff’s Perceptions of the Impact of Integration of PROs into the Clinic Process

Survey results for the clinic staff showed that all 5 agreed that the PRO assessment added value to patient care (Fig. 4B). Clinic staff disagreed whether the PRO assessment saved them time: 2 (40%) agreed, 2 (40%) neither agreed nor disagreed, and 1 (20%) disagreed (Fig. 4B). Additional tasks completed by the on-site research coordinators were estimated to require approximately 4 min per patient and included explaining the procedure to patients, peripheral monitoring to determine when the patient had finished, and tablet computer stewardship and sanitization.

#### Patients’ Perceptions of the Value, Experience, and Barriers of Introducing PROs into Their Care Consultation with the Provider

**Fig. 5:**
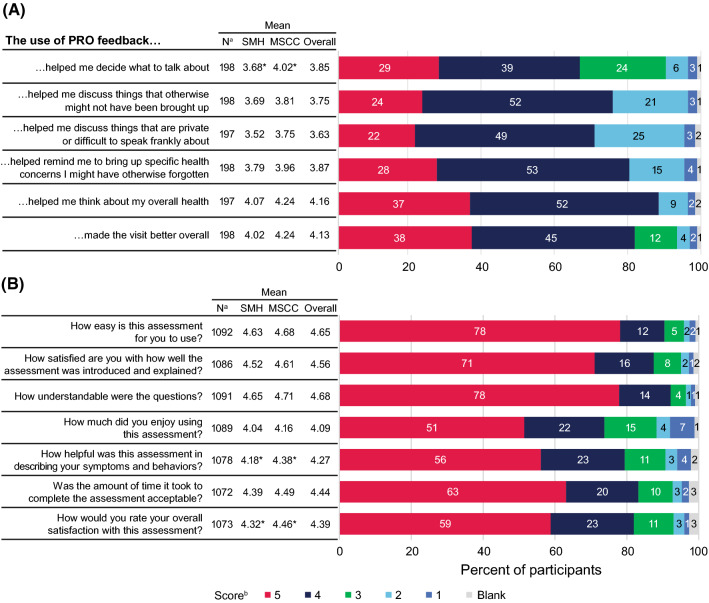
Results of **A** PIPPI (N = 200) and **B** Acceptability E-scale (N = 1102) surveys to assess participants’ perceived impact and acceptability of PROs. ^a^Number of participants who responded to the question. ^b^Participants were asked to score the statements on a scale of 1 to 5. For panel A, 1 represented strong disagreement and 5 represented strong agreement. For panel B, 1 represented a low level of ease, satisfaction, and understanding and 5 represented a high level. *Mean scores significantly different between sites (*P* < 0.05, *t* test). *MSCC* Midway Specialty Care Center, *PIPPI* perceived impact of PROs on patient-provider interaction, *PRO* patient-reported outcomes assessment, *SMH* St Michael’s Hospital

In the post-visit survey, 164/200 (82%) PWH agreed or strongly agreed that the PRO assessment made their visit to the clinic better (Fig. 5A). Most PWH also agreed or strongly agreed that the PRO assessment helped them consider overall health (177/200, 88%), recall health concerns to raise (161/200, 80%), discuss topics that might otherwise not have arisen (152/200, 76%), discuss issues difficult to speak frankly about (142/200, 71%), and decide what to talk about (134/200, 67%). Sixty-five percent (129/198) of PWH surveyed reported that they had discussed the burden of their HIV medication and its impact on their life. Comparison of responses between sites found that patients at MSCC were more likely than patients at SMH to indicate that PROs helped them decide what to talk about (Fig. 5A).

### ‘Adoption’ of PROs by Clinic Personnel

Early and strong leadership endorsement was established through a pre-study site visit, organizational readiness assessment, and joint development of an iterative action plan. This was followed by preparation work by each clinic, communicating a process by which the clinic and each role could benefit (e.g., ensuring the capture of information that would already need to be gathered in routine care, thus addressing multiple needs and saving time overall). The PRO assessment was introduced as a hands-on activity for providers and clinic staff during the initial session; this engaged interest and inspired possibilities for application to their needs/setting.

On the basis of the survey findings, there was 100% adoption among providers and staff of the PRO.

### ‘Implementation’—The Fidelity of PRO Integration, Including Consistency of Delivery, Use as Intended, and the Time and Cost of the Intervention

#### Fidelity of PRO Integration

Fidelity to core elements was complete and consistent, including training completion at sites by all relevant staff, appointment of a champion/coordinator at sites with defined responsibilities, application of the PRO process to eligible patients according to protocol, and use of a results summary within consultations by providers as part of routine care.

#### Provider and Staff Satisfaction with Training and Implementation

All 18 providers and clinic staff who completed the post-training survey agreed or strongly agreed that the objectives of the training were clearly defined; participation and interaction were encouraged; the topics covered were relevant to them; the content was organized and easy to follow; training objectives were met; time allotted for training was sufficient; and the training increased their confidence in their ability to work with PROs. Most (80%) clinic staff surveyed agreed that PRO assessments had integrated well into their clinic flow and that PROs were not difficult to implement (Fig. 4B).

#### Costs of PRO Implementation

As reported by the on-site research coordinators, the costs of implementing the PRO assessment were low in terms of consumables: the initial purchase of tablet computers/iPads (4 at MSCC and 8 at SMH, at a cost of $329 each) and printing costs where the assessment report was delivered on paper. The largest costs were human resources, including time for setup, training, monitoring, and reviewing. Once the PRO program was established, the on-site research coordinators estimated that each PRO assessment took around 4 min of time for administrative staff in total, including patient engagement, monitoring, and tablet computer preparation and sanitization. With an average of 7.3 and 10.7 PRO assessments per day at MSCC and SMH, respectively, this equated to 6% and 9% of the time of a full-time employee.

### ‘Maintenance’—The Extent to Which the Intervention can be Sustained over the Longer Term

The future maintenance or sustainability of the PRO assessment process at the study clinics was regarded as having been influenced positively by several factors, including leadership engagement, early stakeholder involvement, planned integration to support and enhance the service, a careful phased introduction, and the demonstration of good value. There was a desire from both sites to continue with PRO assessments after the end of the study within their own resources.

### Acceptability

Mean scores from patients’ responses to the Acceptability E-scale built into the PRO assessment were all above 4, indicating high levels of satisfaction (Fig. 5B). Most participants enjoyed using the assessment (4.09/5) and found it easy to use (4.65/5), well explained (4.56/5), understandable (4.68/5), and helpful in describing their symptoms and health behaviors (4.27/5). They also found the amount of time taken to complete the PRO assessment to be highly acceptable (4.44/5). The overall mean satisfaction rating was 4.39/5. There were site differences in responses to specific questions. Compared with patients at SMH, patients at MSCC were more likely to find the PRO assessment helpful in describing symptoms and behaviors (4.18 vs. 4.38; *P* < 0.01) and were more likely to be satisfied with the assessment overall (4.32 vs. 4.46; *P* = 0.01; Fig. 5B).

## Discussion

The PRO assessment evaluated in this study improved the frequency of provider identification, documentation, and referral for complex health and behavioral issues, many of which are known to be less observable, underreported, and/or inadequately addressed in consultations. Differences in documentation between the phases without vs with PRO feedback to providers were significant for anxiety and suicidal ideation, both of which were poorly documented during the preparation phase without PRO feedback (24% and 38%, respectively) and shifted to being documented in the majority of cases in the delivery phase with PRO feedback. There were also increases in documentation for dissatisfaction with antiretroviral medication, depression, and experience of psychological violence from an intimate partner. For some domains, documentation was infrequent (approximately one-third or fewer cases) during both phases, regardless of PRO feedback, including sexual risk behavior, hazardous drinking, and substance use. This is in contrast with findings from a similar analysis by Crane et al. (2017) in which documentation of ‘at-risk alcohol use’ and ‘substance use’ increased after implementing provider feedback of PROs, although the differences were not always significant [16]. The impact on referral (where applicable) in our study was smaller than the impact on documentation, with only patients with anxiety being significantly more frequently referred to mental health services. Differences between domains in terms of documentation and referral may be related to provider perception of the importance of different issues, the provider’s comfort with addressing specific issues vs need for additional support, and the availability of appropriate follow-up services. It is notable that approximately one-quarter of participating PWH indicated dissatisfaction with their antiretroviral treatment given that the study was conducted at a time when several once-daily antiretroviral treatment options with low toxicity are available. These results align with data from the Positive Perspectives study, which indicate that many PWH continue to face challenges with their antiretroviral therapy that they may not be raising with their healthcare providers, including difficulties swallowing pills, stress from a daily dosing routine, and a fear of revealing their HIV status by taking pills [24].

The acceptability of the PRO assessment by all stakeholders at the clinics in this study was high and was consistent across all measures employed [17, 25]. In the survey, most providers agreed that PROs added value to the consultation by helping to prioritize discussion topics and identifying topics that would not otherwise have been addressed. Similarly, survey data from non-medical staff showed a high level of agreement on the ease of integration of PROs into clinic flow and the added value for patients. The Acceptability E-scale built into the PRO assessment showed that PWH also found the PROs useful and easy to use, with a mean overall satisfaction rating of 4.39 out of 5.

The use of tablet-based PRO assessments was likely an important factor in the high acceptability of PROs in this study. Patients generally prefer tablet-based over paper-based administration [26, 27], and the ability to integrate skip patterns into the design of the assessment (e.g., not asking nonsmokers about smoking patterns) ensures a manageable time burden on patients and decreases impact on patient flow. Digital administration of PROs has several other advantages, including automated scoring within domains, reducing scoring errors, and the ability to link PRO responses to real-time alerts and to rapidly generate summary results. Additionally, the use of patient portals or URLs has the potential to open access to PROs to a wide range of users, including those with restricted access to a physical clinic. The past decade has seen increasing use of telemedicine in HIV care, and this is likely to be accelerated by the COVID-19 pandemic, which has prompted widespread consideration of more permanent use of remote care delivery methods [28–31]. Challenges to the use of telemedicine in HIV care include concerns over inadequate interpersonal connection between the patient and provider, leading to inability to properly assess complex health issues [28]. To address this concern, it is important to develop strategies that maintain a patient-centered approach to HIV care via telemedicine. The use of digital PROs in this context can reduce reporting bias when in physical healthcare settings, help providers better understand their patients’ needs and health behaviors regardless of distance, and identify those most at risk of loss to follow-up.

A language barrier was the most common reason for patients not engaging with the PRO assessment. This could be relatively easily overcome by implementing translations, which for many validated PRO measures are widely available in multiple languages. This was identified early on and was the reason for the addition of a Haitian Creole version partway through the study, resulting in a decrease in the refusal rates. Using a digital format, there is also the ability to include an audio component for PWH with poor vision or low literacy. A results summary can be generated in the provider’s desired language regardless of the language in which the assessment was administered to the patient.

One strength of this study is that it evaluated the implementation of PROs into daily practice in community-based outpatient HIV clinics and therefore may be applicable to a greater number of settings than previously published data from clinical trials or studies conducted at larger, highly resourced academic centers. A further strength is that data were collected from all salient stakeholders, including non-medical clinic staff for whom the implementation of PROs could have a significant impact. A limitation is that the study was done at only two North American clinics, which were selected to have characteristics increasing the likelihood of successful implementation of PROs into their practice, such as high leadership engagement for implementation. In addition, since PWH who dislike questionnaires were able to opt out of participating, the study population is likely to be representative of patients who tolerate this type of PRO assessment. Nevertheless, the implementation learnings acquired during the study provide a valuable framework from which to develop customized PRO programs for a variety of situations. These learnings have informed the development of a publicly available PRO implementation toolkit for integrating PRO assessments into routine HIV care (available at https://progresshivcare.org/), which was created as part of this study [32].

While this study provides a rich description of lessons learned from the implementation of a web-based PRO into routine HIV care at two outpatient clinics, many questions remain unanswered. Next steps include a focus on the insights of implementation and impact of PROs over time (longitudinal analyses), particularly as it relates to the maintenance phase of RE-AIM and the long-term implementation lessons. As more data accrue, there will be the opportunity to evaluate satisfaction with medications using the HATQoL questions in the context of individual regimens. In addition, as noted in Table 1 and Fig. 2, we have also been conducting provider and patient interviews. This qualitative evaluation will enrich and add context to the quantitative findings presented here. Lastly, the PRO process developed as part of this study is being implemented at a further three sites in the Toronto Ontario HIV Treatment Network in addition to SMH, and there is an intent to extend this to all 21 sites in the network in the future.

In conclusion, in these two North American HIV clinics, providers found PROs with results delivery before patient appointments both useful and acceptable for routine HIV care. PROs may facilitate prioritization of issues to address and expedite identification of sensitive topics, particularly anxiety and suicidal ideation. This may offset additional burden on clinic flow and provider workload. PWH receiving care at the clinics found PROs administered before the appointment to be useful for prioritizing discussion topics with their providers, helping initiate discussion on sensitive issues, and improving comprehensiveness of and satisfaction with care.

## Supplementary Information

Below is the link to the electronic supplementary material.

Supplementary file1 (PDF 102 kb)

Supplementary file2 (PDF 168 kb)

## Data Availability

Anonymized individual participant data and study documents can be requested for further research from www.clinicalstudydatarequest.com.
