# Supplementary material for: Utility and Impact of the Implementation of Same-Day, Self-administered Electronic Patient-Reported Outcomes Assessments in Routine HIV Care in two North American Clinics
Source: AIDS Behav. 2022 Jan 22;26(7):2409–24. doi: 10.1007/s10461-022-03585-w (PMC8783196; doi:10.1007/s10461-022-03585-w)
Supplement: Supplementary file 1 — Supplementary file1 (PDF 102 kb) [file 10461_2022_3585_MOESM1_ESM.pdf]

SUPPLEMENTARY APPENDIX 1

Acceptability E-scale items in patient-reported outcomes (PRO) assessments

Participants were asked to score the following statements on a scale of 1 to 5, with 1 representing a low level of ease, satisfaction, understanding, etc

1. How easy was this assessment for you to use?
2. How satisfied are you with how well the assessment was introduced and explained?
3. How understandable were the questions?
4. How much did you enjoy using this assessment?
5. How helpful was this assessment in describing your symptoms and health behaviors?
6. Was the amount of time it took to complete this assessment acceptable?
7. How would you rate your overall satisfaction with this assessment?

Structured post-training survey questions for all providers

Questions were scored on a 5-point scale (Strongly agree/Agree/Neither agree nor disagree/Disagree/Strongly disagree)

1. The objectives of the training were clearly defined
2. Participation and interaction were encouraged
3. The topics covered were relevant to me
4. The content was organized and easy to follow
5. Training objectives were met
6. Time allotted for training was sufficient
7. The training increased my confidence in my ability to work with PROs

Structured post-interview survey questions for clinical staff

Questions 1 to 6 scored on a 5-point scale (Strongly agree/Agree/Neither agree nor disagree/Disagree/Strongly disagree), question 7 scored on a 4-point scale (All of the time/Most of the time/Some of the time/Never)

The use of PRO assessment feedback:

1. Helped me prioritize what to talk about
2. Helped me address things that might not otherwise have been brought up
3. Led me to initiate discussions that are traditionally more private or difficult for patients to speak frankly about
4. Made my consultation easier
5. Saved me time
6. Added value to the visit overall
7. For the patients from whom PRO is appropriate, to what extent have you adopted the use of PRO feedback into your practice

Structured post-interview survey questions for non-medical staff

All questions scored on a 5-point scale (Strongly agree/Agree/Neither agree nor disagree/Disagree/Strongly disagree)

PRO assessments:

1. Were difficult to implement into care
2. Have integrated well into clinic flow
3. Add value to patient care
4. Make it easier to do my job
5. Saved me time
6. Are well tolerated by patients

Structured survey questions for patients

Questions with options for yes/no/don't know

1. Did you and your provider discuss the results of your iPad survey today?

Did you and your provider discuss:

2. Quality of life
3. Depression
4. Burden and impact that medicines have on my life
5. Taking HIV medications as prescribed
6. Cigarette use
7. Alcohol use
8. Drug use
9. Sexual behavior/safer sex
10. Partner violence

Questions scored on a 5-point scale (Strongly agree/Agree/Neither agree nor disagree/Disagree/Strongly disagree)

PRO assessments:

1. Helped me decide what to talk about
2. Helped me discuss things that otherwise might not have been brought up
3. Helped me discuss things that are private or difficult to speak frankly about
4. Helped remind me to bring up specific health concerns I might have otherwise forgotten
5. Helped me think about my overall health
6. Made the visit better overall
