# Supplementary material for: Utility and Impact of the Implementation of Same-Day, Self-administered Electronic Patient-Reported Outcomes Assessments in Routine HIV Care in two North American Clinics
Source: AIDS Behav. 2022 Jan 22;26(7):2409–24. doi: 10.1007/s10461-022-03585-w (PMC8783196; doi:10.1007/s10461-022-03585-w)

SUPPLEMENTARY FIGURE 1. Reasons for refusal/non-completion of PRO assessments (n=181). PRO, patient-reported outcomes assessment.

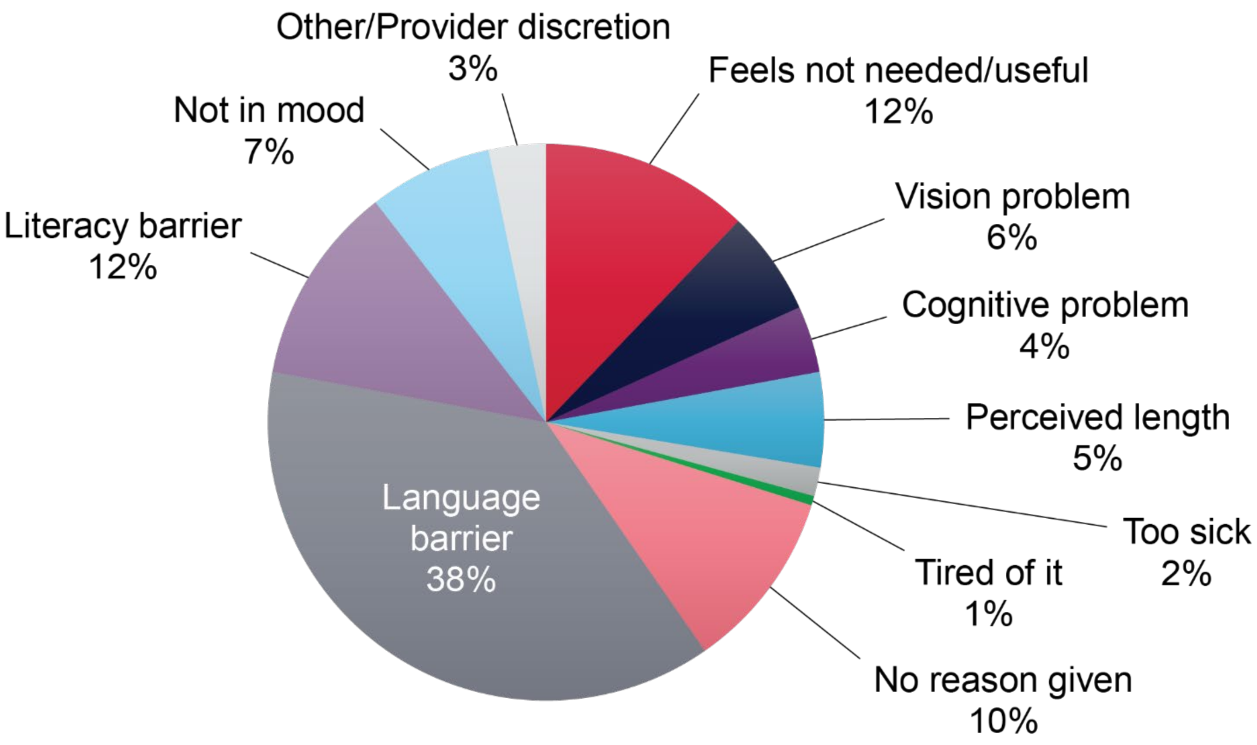

Supplement: Supplementary file 2 — Supplementary file2 (PDF 168 kb) [file 10461_2022_3585_MOESM2_ESM.pdf]
